# C-reactive protein during pregnancy and in the early postpartum predicts adverse metabolic health outcomes at 1 year postpartum in women with gestational diabetes

**DOI:** 10.1186/s12933-023-02034-9

**Published:** 2023-10-27

**Authors:** Dan Yedu Quansah, Antje Horsch, Leah Gilbert, Marc Y. Donath, Jardena J. Puder, the MySweetheart research group, Amar Arhab, Pascal Bovet, Arnaud Chiolero, Stefano Di Bernardo, Adina Mihaela Epure, Sandrine Estoppey Younes, Leah Gilbert, Justine Gross, Antje Horsch, Stefano Lanzi, Seyda Mayerat, Yvan Mivelaz, Jardena J. Puder, Dan Yedu Quansah, Jean-Benoit Rossel, Nicole Sekarski, Umberto Simeoni, Bobby Stuijfzand, Yvan Via.

**Affiliations:** 1 Obstetric Service, Department Woman-Mother-Child, Lausanne University Hospital, Rue du Bugnon 21, CH-1011 Lausanne, Switzerland; 2 Institute of Higher Education and Research in Healthcare (IUFRS), University of Lausanne, Lausanne, Switzerland; 3 Neonatalogy Service, Department Woman-Mother-Child, Lausanne University Hospital, Lausanne, Switzerland; 4 Endocrinology, Diabetes and Metabolism, University Hospital Basel, and Department of Biomedicine, University of Basel, Basel, Switzerland

**Keywords:** Gestational diabetes, C-reactive protein, Perinatal period, Metabolic outcomes, Postpartum, Body fat, Insulin resistance, Insulin secretion

## Abstract

**Background:**

Women with gestational diabetes mellitus (GDM) have higher insulin resistance and/or reduced secretion, an increased risk of future diabetes and cardiovascular disease, which may be due to a pathological activation of the innate immune system. C-reactive protein (CRP) is induced by inflammatory cytokines and reflects innate immune activity. We investigated the prospective associations between CRP during the perinatal period with adverse metabolic outcomes at 1 year postpartum in women with previous GDM.

**Methods:**

We analyzed data from the MySweetheart trial that included 211 women with GDM at 28–32 weeks gestational age (GA). CRP was measured during  pregnancy at 28-32 weeks GA, at 6–8 weeks and at 1 year postpartum. Metabolic outcomes at 1 year postpartum included weight, total and central body fat, measures of insulin resistance and secretion and presence of the metabolic syndrome (MetS). A 75 g oral glucose tolerance test was performed to measure glucose and insulin values every 30 min over 2 h to calculate indices of insulin resistance (MATSUDA, HOMA-IR) and of absolute (AUC_ins/glu_, HOMA-B) and insulin resistance-adjusted insulin secretion (ISSI-2).

**Results:**

CRP during pregnancy and at 6–8 weeks postpartum predicted increased weight, body fat and visceral adipose tissue (VAT), insulin resistance (higher HOMA-IR, lower MATSUDA), absolute insulin secretion (HOMA-B, AUC_ins/glu_), a reduced adjusted insulin secretion (ISSI-2) and a higher prevalence of the MetS at 1 year postpartum (all p ≤ 0.036). These relationships particularly those concerning CRP during pregnancy, were independent of weight ( for VAT, insulin resistance and secretion indices, MetS; all p ≤ 0.032) and of body fat ( for VAT, MATSUDA, MetS; all p ≤ 0.038).

**Conclusion:**

CRP during pregnancy and in the early postpartum predicted an adverse cardio-metabolic profile in women with prior GDM at 1 year postpartum independent of weight. The prospective association of CRP with increased insulin resistance and reduced adjusted insulin secretion hint to the role of inflammation in the development of impaired metabolism after GDM and could be used as an early marker for risk stratification.

**Supplementary Information:**

The online version contains supplementary material available at 10.1186/s12933-023-02034-9.

## Background

Gestational diabetes mellitus (GDM) is associated with increased risks of metabolic complications such as obesity, diabetes and cardiovascular disease (CVD) in the postpartum, the latter even in the absence of diabetes [1, 2]. Compared to women without GDM, the risk for diabetes is explained by their increased insulin resistance and decreased insulin secretion [3]. In this context, the perinatal period is a critical time to identify women at higher risk for metabolic complications in this population to provide early interventions.

Chronic inflammation is implicated as one of the underlying disorders associated with insulin resistance, development of diabetes, and CVD [4]. It has been shown that C-reactive protein (CRP), a systemic acute phase protein, is a predictor of CVD at levels > 2 mg/l [5] and a marker of insulin resistance [6]. CRP is amongst others regulated by the pro-inflammatory cytokine IL-1β [7], IL-6 and tumor necrosis factor-alpha (TNF-α) [8], and reflects innate immune activity and represents a marker of chronic systemic sub-clinical inflammation [9]. The link between CRP and the acute-phase response [10], its contribution to impaired insulin signaling pathway [11], and in part, its links with obesity-driven systemic inflammation can explain the association with insulin resistance [12]. A recent preclinical study showed that CRP can reduce insulin secretion by influencing beta-cell function directly in isolated mouse islet cells [13]. Notwithstanding, human data on CRP and insulin secretion are limited, as only one cross-sectional study found a link between CRP and impaired insulin secretion in a non-diabetes population [14]. However, it is likely that CRP is controlled by levels of cytokines including IL-1β, TNF-α and IL-6 that have parallel effects on insulin secretion and action rather than direct effects of CRP.

In pregnancy, CRP levels are generally increased and even more so in women with GDM [15, 16]. Around four months postpartum, CRP levels correlate with hepatic insulin resistance in women with GDM [15]. Retrospective cohorts of women in the postpartum show that those with previous GDM who are obese or have features of the metabolic syndrome (MetS), have higher CRP levels compared to those without previous GDM [17, 18]. The role of inflammation in GDM is supported by an animal model in which inhibition of IL-1β improved glycaemia [19]. Excessive weight gain, weight retention and poor glucose control that characterize GDM can lead to an increase in inflammatory parameters that precedes insulin resistance, diabetes, and CVD outcomes.

There is a lack of longitudinal data regarding CRP and measures of hepatic and overall insulin resistance in women with GDM. Furthermore, it remains unclear whether the relationship between CRP and insulin resistance is independent of weight, body fat or fat-free mass in women with GDM. Whereas fat mass has been shown to increase insulin resistance, fat-free mass is protective. Particularly, there is a lack of knowledge if CRP can also impact on insulin secretion in women with GDM. Given the important role of CRP regarding body fat, insulin resistance, diabetes and CVD, longitudinal studies could assist to better understand the underlying mechanisms and help to guide early risk stratification and intervention for this young but high-risk population.

The aim of the study was to investigate the prospective associations between CRP during pregnancy and in the early postpartum with adverse metabolic outcomes including body fat, insulin resistance and secretion at 1 year postpartum in women with GDM and to determine the extent to which potential relationships are independent of body weight or fat or fat-free mass.

## Methods

This current study is a secondary analysis of the MySweetheart trial (NCT02872974). The trial tested the effect of an interdisciplinary pre- and postpartum lifestyle and psychosocial intervention in women with GDM. The study protocol was approved by the Human Research Ethics Committee of the Canton de Vaud (Study number 2016–00745). The detailed study protocol has been previously described [20]. Out of the 211 women included at baseline at 28–32 weeks gestational age (GA; n = 105 in the intervention and n = 106 in the usual care group), 20 women were excluded from this analysis at 6–8 weeks postpartum (191 women at 6–8 weeks postpartum) and 34 at 1 year postpartum (157 women at 1 year postpartum). Of the 54 women excluded, 87% (n = 47/54) were lost to follow-up, 13% (n = 7/54) had new pregnancies before the 1 year postpartum. All included participants had data for CRP at all three time points. The values of predictors and outcomes were similar in the intervention and control groups, so we pooled participants together and all analyses were adjusted for group allocation (see statistical analyses section).

### GDM diagnosis, management, and patient follow-up

Women were diagnosed with GDM during pregnancy at 28–32 weeks GA based on a one-step 75 g oral glucose tolerance test, in accordance with the International Association of Diabetes and Pregnancy Study Groups (IADPSG) and the American Diabetes Association (ADA) guidelines [21, 22] We followed-up women in the usual care group according to the ADA and the Endocrine Society guidelines [22, 23]. They were first seen at 28–32 weeks GA by a physician, or diabetes-specialist nurse who then followed them until delivery. Women received information on GDM, dietary advice by a dietician and tailored recommendations regarding lifestyle changes and gestational weight gain (GWG) based on the Institute of Medicine (IOM) recommendations [24] and were advised to engage in physical activity. Overall, we placed a strong focus on behavioral changes and treatment with insulin, or very rarely with metformin was introduced when glucose values remained above targets according to Swiss guidelines [25]. On top of the usual care, the full intervention program consisted of five individual interdisciplinary lifestyle sessions during pregnancy and four sessions in the postpartum, a peer support group workshop both in pregnancy and postpartum, and a bimonthly lifestyle coach support mostly through telemedicine. It focused on tailored strategies to improve diet, physical activity, mental health, and social support, and to improve adherence to GWG and weight retention recommendations. The detailed description of the MySweetheart trial has been previously described [26].

## Measures

### Measurement of C-reactive protein (CRP) and cytokines

We measured CRP at 28–32 weeks GA (baseline), at 6–8 weeks and 1 year postpartum. CRP was analysed at the Lausanne University Hospital in serum aliquots using a latex-enhanced immunoturbidimetric assay on a Cobas 8000 autoanalyser (Roche Diagnostics, Mannheim, Germany) with assay characteristics as reported by the manufacturer ((LOD (0.5–1), CV intra (3.7% at 0.840 mg/l) and CV total (inter) (4.0% at 0.840 mg/l)). We also measured IL-6 (U-PLEX Human IL-6 Antibody Set (LLOD: 0.27–1.3) and CV intra (3.0% at 855 pg/ml) and CV total (inter) (5.9% at 855 pg/ml) and TNF-α (U-PLEX Human TNF-α Antibody Set ((LLOD: 0.36–0.76), and CV intra (2.9% at 1560 pg/ml) and CV total (inter) (52% at 1560 pg/ml) at 28–32 weeks GA, at 6–8 weeks and at 1-year postpartum using ELISA according to the manufacturer’s instructions.

### Metabolic health variables

Pre-pregnancy weight was extracted from participants’ medical charts or, if missing, was self-reported. We measured weight and height at 28–32 weeks GA, and weight at the end of pregnancy, at 6–8 weeks and 1 year postpartum using electronic scales (Seca^®^). Information on the need for glucose-lowering medical treatment (use of insulin and/or metformin) during pregnancy was extracted from maternal medical records. GWG was defined as the difference in pre-pregnancy weight and weight at the end of pregnancy and weight retention was calculated as the difference in pre-pregnancy weight and weight at either 6–8 weeks or 1 year postpartum. Body fat and fat-free mass were assessed at 28–32 weeks GA, at 6–8 weeks and 1 year postpartum using Bioelectrical Impedance analysis (BIA) (Akern BIA 101) [27]. We assessed visceral adipose tissue (VAT) at 1-year postpartum using Dual-Energy X-ray Absorptiometry (DXA) in 109 women who signed an additional consent form for this procedure. Metabolic syndrome (MetS) status was defined according to the International Diabetes Federation guidelines, which is based on either waist circumference > 80 cm or BMI ≥ 30 kg/m^2^ and at least two of the following cut-offs: triglycerides ≥ 1.7 mmol/l, HDL < 1.3 mmol/l, blood pressure ≥ 130/85 mmHg, FPG ≥ 5.6 mmol/l or type 2 diabetes mellitus [28]. As women are in a perinatal context, we use the definition with either the BMI or the waist circumference cut-off.

HbA1c was measured with a chemical photometric method (conjugation with boronate-Afinion^®^) at 28–32 weeks GA, and with a High Performance Liquid Chromatography method (HPLC) in the postpartum according to international guidelines [29]. At baseline, we performed fasting measures of glucose and insulin and at 6–8 weeks and 1-year postpartum, we additionally performed a 75 g oral glucose tolerance test (oGTT). We defined prediabetes (FPG 5.6–6.9 mmol/l or HbA1c 39-46 mmol/mol (5.7–6.4%) or 2 h glucose 7.8–11.0 mmol/l) and diabetes (FPG ≥ 7.0 mmol/l, 2 h glucose ≥ 11.1 mmol/l or HbA1c ≥ 48 mmol/mol (6.5%)) according to the ADA criteria.

### Insulin secretion/sensitivity indices

During the oGTT, we measured glucose and insulin values at fasting, and every 30 min over 2 h to calculate insulin secretion/sensitivity indices. Insulin was measured using the electrochemical luminescence immunoassay (ECLIA) on Cobas e 800 autoanalyzer (Roche Diagnostics, Mannheim, Germany). The Homeostatic Model Assessment for Insulin Resistance (HOMA-IR) was used as a measure of insulin sensitivity [30]. Whole body insulin sensitivity was estimated with the Matsuda index [31]. Insulin resistance-adjusted insulin secretion was assessed using Insulin Secretion-Sensitivity Index-2 (ISSI-2), also known as disposition index, expressed as the product of Matsuda index and AUC_ins/gluc_ [32]. Absolute insulin secretion was estimated using Area under the Curve (AUC_ins/glu_) according the trapezoidal rule [31], while HOMA of b-cell index (HOMA-B) was computed as 20 × fasting insulin (μIU/ml)/ fasting glucose (mmol/ml)− 3.5 [33].

### Socio-demographic and medical characteristics

Information on maternal socio-demographic characteristics including age, nationality/ethnic origin and educational level were collected at baseline. Data on medical characteristics including previous history of GDM, family history of diabetes, gravida, and parity as well as of breastfeeding were extracted from participants’ medical charts if available or were obtained from participants during the visits.

## Statistical analyses

All statistical analyses were performed with Stata/SE 15.1 (StataCorp LLC, TX, USA). Socio-demographic and medical characteristics were presented as either means (± standard deviation) or in frequency and percentages (%) (Table 1). Dependent variables included weight, body fat (BIA), VAT (DXA), MetS (BMI and WC), fasting glucose, 2 h glucose after oGTT, HbA1c, indices of insulin resistance (HOMA-IR, MATSUDA) and insulin secretion (ISSI-2, AUC_ins/glu_ and HOMA-B) measured during pregnancy and in the postpartum. Predictor variables included CRP at baseline, at 6–8 weeks and 1 year postpartum. Both outcomes and predictors were normally distributed. We used linear regression models (logistic regression for binary variables) to investigate the prospective associations between CRP at baseline and at 6–8 weeks postpartum with metabolic outcomes at 1 year postpartum. In model 1, we adjusted for group allocation, in model 2, for group allocation, and for the following confounders regarding medical history and diabetes risk: GA, age, previous history of GDM, family history of diabetes, breastfeeding at 6–8 weeks postpartum, as well as for weight (at the prediction time point) and in model 3, for group allocation, the above-mentioned medical history confounders as well as for body fat (at the prediction time point). We also determined the cross-sectional associations between CRP and metabolic variables at baseline, at 6–8 weeks and at 1 year postpartum using the same regression models. When body fat or weight were the outcome variable, we did not adjust for the respective variable in the regression models. All reported beta-coefficients were standardized. In all analyses, both predictors and outcomes were similar in the intervention and usual care groups. The result and effect sizes of the relationship between CRP and metabolic outcomes were similar when we restricted the analyses only to the control group, so we pooled participants in both groups to increase the sample size and always adjusted for group allocation in all analyses.

**Table 1:** Baseline maternal socio-demographic and clinical characteristics of study participants

Variable	Mean ± SD
*N*	211
Age (year)	33.8 ± 4.4
GA at 28–32 week GA (weeks)	28.8 ± 2.4
BMI at 28–32 week GA (kg/m^2^)	29.6 ± 5.0
BMI at 28–32 week GA (kg/m^2^) (IQR)	29.1 (6.8)
GWG up to at 28–32 week GA (kg)	10.2 ± 5.8
Nationality/Ethnicity (n, %)	
Switzerland	62 (32.4)
Rest of Europe and North America	83 (43.4)
Asia and Oceania	23 (12.0)
Africa	14 (7.3)
Latin America	7 (3.7)
Others	2 (1.0)
Education level^a^ (n, %)	
Compulsory school incomplete^b^	2 (1.1)
Compulsory school achieved	23 (13.0)
High school	19 (10.7)
General and vocational education	42 (23.7)
University	91 (51.4)
Glucose-lowering treatment in pregnancy (n, %)	
Yes	90 (42.6)
No	121 (57.4)
Parity (n, %)	
0	120 (56.9)
1	57 (27.0)
2	18 (8.5)
≥ 3	16 (7.6)
Gravida (n, %)	
1	88 (41.7)
2	50 (23.7)
≥ 3	73 (34.6)
GDM in previous pregnancy^c^ (n, %)	
Yes	25 (11.8)
No	186 (88,2)
Family history of diabetes^d^ (n, %)	
Yes	136 (64.4)
No	75 (35.6)

GDM denotes gestational diabetes mellitus; GA denotes gestational age; SD denotes standard deviation; GA denotes gestational age; BMI denotes body mass index, GWG denotes gestational weight gain; IQR denotes Interquartile range

^a^34 participants had missing data on education

^b^In Switzerland, compulsory schooling lasts eleven years

^c^Only for women who had at least one previous pregnancy

^d^Family history of diabetes consists of those with first degree (e.g., mother, father, brother, sister, daughter, son) and second degree (at least 25% of genetic link that included grandparents, grandchildren, nephews, niece, half-brother, and half-sister) relationship of the participant

All values are expressed as mean and standard deviations or n, %

In a supplementary analysis, we determined the differences in CRP, TNF-α and IL-6 levels at baseline, and in the postpartum in all participants and according to pre-pregnancy BMI categories (Additional file 1: Table S1). In an additional supplementary longitudinal analysis, we adjusted for fat-free mass in addition to group allocation and the medical history confounders (Additional file 1: Table S3). Additional analyses also included the relationship between CRP during pregnancy and at 6–8 weeks postpartum with metabolic health outcomes at 1-year postpartum, adjusted for changes in weight or fat mass (Additional file 1: Table S5). We also investigated the metabolic outcomes during pregnancy and postpartum according to pre-pregnancy BMI categories (Additional file 1: Table S2). In addition, we investigated the longitudinal associations between IL-6 and TNF-alpha during pregnancy and at 6–8 weeks postpartum with metabolic health outcomes at 1-year postpartum (Additional file 1: Table S4).

For all analyses, we adjusted each time for only one body composition measure in the same analyses due to modestly high correlations between weight, body fat and fat free mass (r = 0.72–0.94) to avoid collinearity. All statistical significances were two sided and accepted at p < 0.05.

## Results

**Fig. 1:**
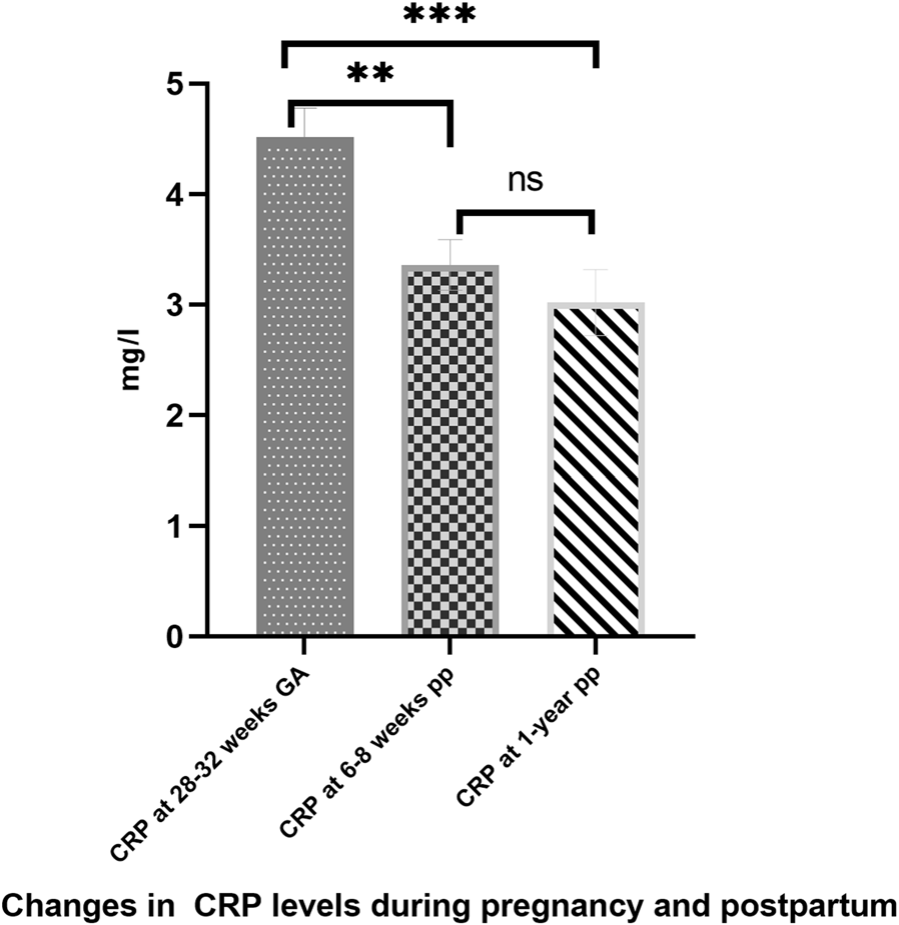
Changes in C-reactive protein (CRP) levels during pregnancy and in the postpartum study participants. CRP at 28–32 weeks GA was significantly higher than levels at 6–8 weeks and 1 year postpartum. The differences between CRP at 6–8 weeks and 1 year postpartum was not significant. GA denotes gestational age; ns denotes not significant; **denotes p < 0.001, ***denotes p < 0.0001

Two hundred and eleven (211) women were included at the baseline visit at a mean GA of 28.8 ± 2.4 weeks. Their pre-pregnancy BMI was 25.8 ± 5.4 kg/m^2^. About 43% of women received glucose lowering-medical treatment (insulin, very rarely metformin (n = 8/211)) during pregnancy (Table 1). No glucose-lowering treatment was given in the postpartum. At 6–8 weeks and 1 year postpartum 75% (159/211) and 20% (32/159) of women in our cohort were breastfeeding. Mean CRP values at 28–32 weeks GA (baseline), 6–8 weeks, and 1 year postpartum were 4.5 ± 3.7 mg/l, 3.4 ± 3.2 mg/l and 3.0 ± 3.8 mg/l respectively (p < 0.001, Additional file 1: Table S1, Fig. 1). Mean weight and BMI at 1 year postpartum were 72.4 ± 16.2 kg and 26.8 ± 4.5 kg/m^2^ and the prevalence of the MetS (“methods” section [28]) was 33.2%. The prevalence of prediabetes and diabetes at 1 year postpartum were 35% (n = 55/157) and 3.2% (n = 10/157) respectively (Additional file 1: Table S2). Data for body fat, glycemic values and different measures of insulin resistance and secretion at the three time points are shown in the Additional file 1: Table S2.

### Longitudinal associations between CRP metabolic health outcomes at 1-year postpartum

Table 2 shows the longitudinal associations between CRP at 28–32 weeks GA (baseline) and at 6–8 weeks postpartum with metabolic health outcomes at 1 year postpartum. CRP during pregnancy predicted increased weight, body fat (BIA), VAT and MetS (according to either BMI or WC) at 1 year postpartum (all p ≤ 0.001). These associations (body fat, VAT, MetS) were independent of medical history confounders (GA age, previous history of GDM, family history of diabetes, breastfeeding) as well as of weight during pregnancy (all p ≤ 0.041). The associations between VAT and MetS-WC were also independent of these confounders as well as of body fat (both p ≤ 0.038). Regarding glycemic parameters, CRP during pregnancy was associated with increased fasting glucose (p = 0.036) but not HbA1c or post-oGTT glucose. It predicted higher insulin resistance (higher HOMA-IR, lower MATSUDA), and increased absolute insulin secretion (AUC_ins/glu_, HOMA-B), but lower insulin resistance-adjusted insulin secretion (relative insulin secretion, ISSI-2) at 1 year postpartum (all p ≤ 0.036). These associations remained significant after adjustment for confounders and for weight (all p ≤ 0.032), while the ones with both indices of insulin resistance also persisted after further adjusting for body fat (p ≤ 0.001).

**Table 2:** Longitudinal associations between CRP during pregnancy and at 6–8 weeks postpartum with metabolic health outcomes at 1 year postpartum

Variable	Model 1			Model 2			Model 3		
	β-coefficient	95% CI	p value	β-coefficient	95% CI	p value	β-coefficient	95% CI	p value
CRP at 28–32 weeks GA									
Weight (kg)^bc^	1.70	1.02, 2.38	** < 0.001**	1.70	1.02, 2.38	** < 0.001**	NA	NA	NA
Body fat (kg)^c^	1.32	0.89, 1.75	** < 0.001**	0.35	0.12, 0.57	** < 0.003**	NA	NA	NA
Visceral adipose tissue (kg)	0.05	0.02, 0.07	** < 0.001**	0.02	0.002, 0.04	**0.024**	0.01	0.001, 0.07	**0.019**
MetS-WC, yes^a^	1.22	1.12, 1.34	** < 0.001**	1.16	1.05, 1.28	**0.002**	1.12	1.06, 1.26	**0.038**
Fasting glucose (mmol/l)	0.03	0.001, 0.05	**0.036**	0.01	**− **0.01, 0.04	0.220	0.02	**− **0.01, 0.05	0.251
2 h glucose (mmol/l)	0.03	**− **0.04, 0.11	0.435	0.008	**− **0.07, 0.10	0.744	0.08	**− **0.08, 0.10	0.847
HbA1c (%)	0.01	**− **0.01, 0.04	0.326	0.005	**− **0.02, 0.03	0.700	0.04	**− **0.03, 0.03	0.749
HOMA-IR	0.20	0.10, 0.31	** < 0.001**	0.19	0.09, 0.29	** < 0.001**	0.16	0.09, 0.30	** < 0.001**
MATSUDA index	**− **0.28	**− **0.42, **− **0.15	** < 0.001**	**− **0.14	**− **0.26, **− **0.01	**0.032**	**− **0.28	**− **0.40, **− **0.16	** < 0.001**
ISSI**-**2	**− **0.05	**− **0.09, **− **0.02	**0.002**	**− **0.05	**− **0.09, **− **0.02	**0.002**	**− **0.02	**− **0.06, 0.01	0.305
AUC_ins/glu_	0.02	0.009, 0.03	**0.002**	0.02	0.009, 0.04	**0.002**	0.04	**− **0.01, 0.02	0.553
HOMA**-**B	2.63	1.23, 4.03	**0.016**	2.20	1.35, 4.25	**0.026**	0.25	**− **1.2, 1.51	0.691
CRP at 6–8 weeks pp									
Weight (kg)^bc^	1.60	0.89, 2.31	** < 0.001**	1.60	0.89, 2.31	** < 0.001**	NA	NA	NA
Body fat (kg)^c^	1.21	0.75, 1.67	** < 0.001**	0.21	0.004, 0.41	**0.046**	NA	NA	NA
Visceral adipose tissue (kg)	0.06	0.04, 0.09	** < 0.001**	0.02	0.002, 0.04	**0.027**	0.02	0.003, 0.03	**0.019**
MetS-WC, yes^a^	1.15	1.05, 1.26	**0.003**	1.09	0.99, 1.21	0.073	1.09	0.98, 1.20	0.095
Fasting glucose (mmol/l)	0.01	**− **0.01, 0.04	0.379	0.003	**− **0.03, 0.03	0.844	**− **0.01	**− **0.03, 0.03	0.927
2 h glucose (mmol/l)	0.02	**− **0.06, 0.10	0.634	**− **0.008	**− **0.09, 0.08	0.855	**− **0.04	**− **0.09, 0.08	0.925
HbA1c (%)	0.04	**− **0.02, 0.02	0.970	**− **0.01	**− **0.04, 0.02	0.393	**− **0.01	**− **0.04, 0.02	0.446
HOMA-IR	0.21	0.11, 0.31	** < 0.001**	0.11	0.02, 0.20	**0.022**	0.11	0.01, 0.21	**0.024**
MATSUDA index	**− **0.27	**− **0.41, **− **0.11	**0.001**	**− **0.29	**− **0.45, **− **0.14	** < 0.001**	**− **0.14	**− **0.19, **− **0.09	**0.001**
ISSI-2	**− **0.06	**− **0.10, **− **0.01	**0.010**	**− **0.02	**− **0.07, 0.02	0.318	**− **0.03	**− **0.07, 0.02	0.307
AUC_ins/glu_	0.02	0.005, 0.03	**0.007**	0.009	**− **0.004, 0.02	0.172	0.09	**− **0.04, 0.02	0.176
HOMA**-**B	2.82	1.52, 4.12	**0.001**	1.44	0.29, 2.60	**0.015**	1.41	0.25, 2.58	**0.017**

CRP denotes C-reactive protein; GA denotes gestational age; MetS denotes metabolic syndrome; BMI denotes Body Mass Index; Mets-WC denotes metabolic syndrome based on waist circumference; pp denotes postpartum; NA denots not applicable; HbA1c denotes glycated hemoglobin; HOMA-IR denotes Homeostatic Model Assessment for Insulin Resistance; ISSI-2 denotes; Insulin Secretion-Sensitivity Index-2; AUCins/glu denotes Area under the Curve; HOMA-B denotes HOMA of b-cell index. Model 1: adjusted for group allocation. Model 2: Adjusted for group allocation, gestational age, age, previous history of GDM, family history of GDM, breastfeeding, weight at the prediction time point. Model 3: adjusted for group allocation, gestational age, age, previous history of GDM, family history of GDM, breastfeeding and body fat at the prediction time point.

^a^Estimates are from logistic regression analyses (Odds ratio and 95% CI)

For these variables, the associations were not adjusted for ^b^weight or ^c^body fat (only adjusted for age).

Bold p-values indicates significant p-values

**Fig. 2:**
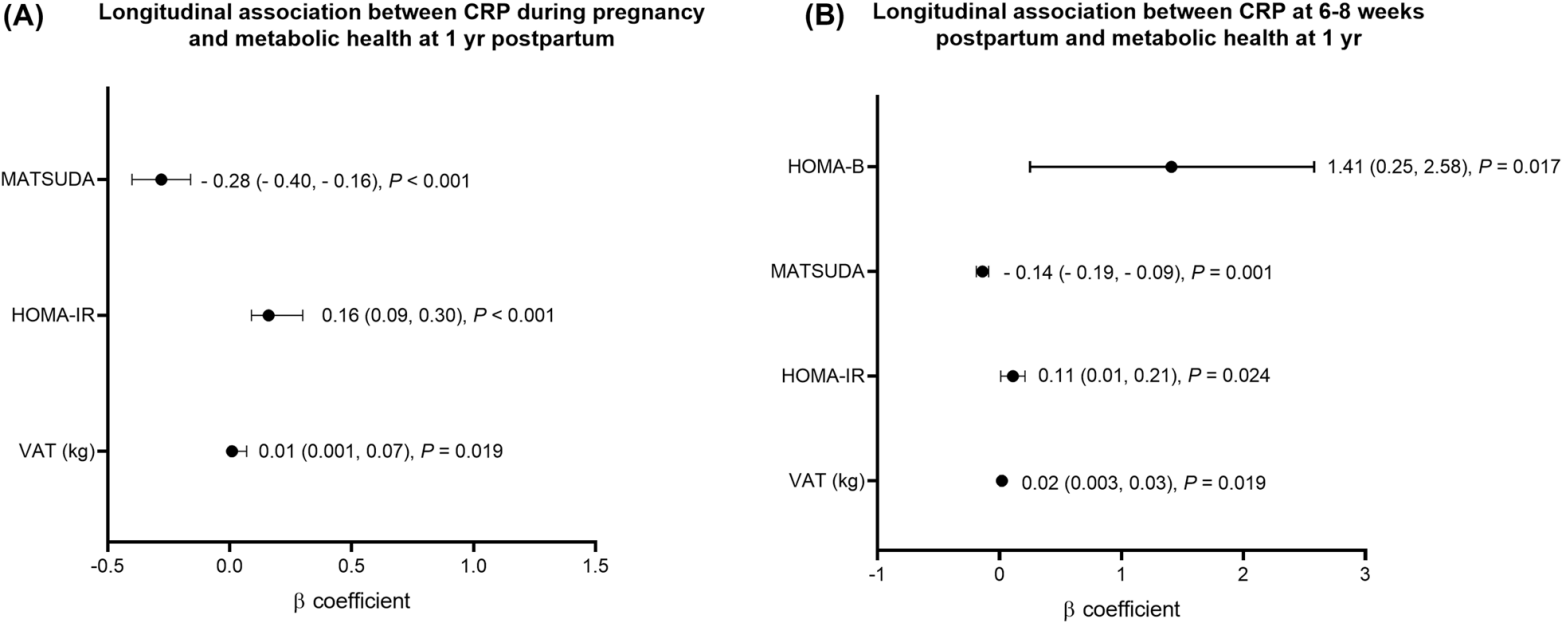
Longitudinal relationships between CRP and metabolic health outcomes at 1-year postpartum in women with gestational diabetes mellitus. **A** Associations between CRP at 28–32 weeks GA and VAT, HOMA-IR, and MATSUDA at 1 year postpartum and **B** Associations between CRP at 6–8 weeks postpartum VAT, HOMA-IR, MATSUDA, and HOMA-B at 1 year postpartum. All analyses were adjusted for GA, age, previous history of GDM, family history of diabetes and of body fat

CRP at 6–8 weeks postpartum was associated with increased weight, body fat, VAT and MetS (BMI and WC) at 1 year postpartum (all p ≤ 0.03). The association with body fat was independent of the medical history confounders and of weight (p = 0.046), while that of VAT was independent of these confounders, weight, or body fat (both p ≤ 0.027). CRP in the early postpartum predicted higher insulin resistance (higher HOMA-IR, lower MATSUDA) and absolute insulin secretion (HOMA-B and AUC_ins/glu_) but reduced relative insulin secretion (ISSI-2) at 1 year postpartum (p ≤ 0.010). The associations with increased insulin resistance (HOMA-IR and MATSUDA) and absolute insulin secretion (HOMA-B) remained significant after further adjusting for confounders and weight or body fat (all p ≤ 0.024). In the postpartum, CRP was not associated with fasting or post-oGTT glucose or HbA1c. Figure 2 summarizes the significant longitudinal associations between CRP both at baseline (A) and at 6–8 weeks postpartum (B) and metabolic health outcomes at 1 year postpartum after adjustment for confounders, and for body fat. We observed similar results when we adjusted for changes in weight or body fat between baseline and 1-year postpartum to account for the time-varying confounding effect of weight or body fat in our longitudinal analyses (Additional file 1: Table S5).

### Cross-sectional associations between CRP and metabolic health outcomes

We also investigated the cross-sectional associations between CRP with metabolic variables during pregnancy and in the postpartum (Table 3). CRP during pregnancy correlated with increased weight, body fat and MetS-BMI (all p ≤ 0.004). It also correlated with increased fasting glucose, HbA1c, increased insulin resistance (HOMA-IR) and absolute secretion (HOMA-B). Regarding cross-sectional associations in the early postpartum (Table 3), CRP correlated with higher weight and body fat, increased insulin resistance (higher HOMA-IR and lower MATSUDA), absolute insulin secretion (HOMA-B and AUCins/glu) and reduced relative insulin secretion (ISSI-2) (all p ≤ 0.009). At 1 year postpartum, CRP correlated with weight, body fat and indices of insulin resistance and secretion (all p ≤ 0.014). Cross-sectional associations were in part independent of medical history  confounders and of weight or, more rarely, body fat.

**Table 3:** Cross-sectional associations between CRP and metabolic health outcomes during pregnancy and in the postpartum

Variable	Model 1			Model 2			Model 3		
	β	95% CI	p value	β	95% CI	p value	β	95% CI	p value
CRP at 28–32 weeks GA	*Insulin resistance and secretion variables during pregnancy*								
Weight (kg)^bc^	1.68	1.18, 2.17	** < 0.001**	1.67	1.2, 2.20	** < 0.001**	NA	NA	NA
Body fat (kg)^c^	1.24	0.94, 1.55	** < 0.001**	0.28	0.15, 0.41	** < 0.001**	NA	NA	NA
MetS-BMI, yes^a^	1.12	1.03, 1.22	**0.006**	1.02	0.90, 1.11	0.965	0.98	0.87, 1.09	0.772
Fasting glucose (mmol/l)	0.02	0.008, 0.04	**0.004**	0.02	0.009, 0.04	**0.004**	0.06	**− **0.02, 0.02	0.995
HbA1c (%)	0.01	0.004, 0.02	**0.007**	0.01	0.007, 0.03	**0.001**	0.09	**− **0.03, 0.02	0.164
HOMA-IR	0.16	0.05, 0.26	**0.003**	0.15	0.05, 0.26	**0.004**	0.002	-0.08, 0.09	0.960
HOMA-B	1.99	0.69, 3.2	**0.003**	0.21	**− **1.06, 1.48	0.742	**− **0.16	**− **1.41, 1.01	0.792
CRP at 6–8 weeks pp	*Insulin resistance and secretion variables at 6–8 weeks postpartum*								
Weight (kg)^bc^	1.48	0.88, 2.09	** < 0.001**	1.48	0.87, 2.08	< 0.001	NA	NA	NA
Body fat (kg)^c^	1.06	0.66, 1.45	** < 0.001**	0.16	0.03, 0.29	**0.015**	NA	NA	NA
MetS-BMI, yes^a^	1.10	0.97, 1.25	0.107	0.98	0.83, 1.15	0.818	0.92	0.76, 1.11	0.421
Fasting glucose (mmol/l)	0.01	**− **0.01, 0.04	0.228	**− **0.005	**− **0.03, 0.02	0.686	**− **0.006	**− **0.03, 0.02	0.665
HbA1c (%)	0.01	**− **0.007, 0.03	0.060	0.02	0.0005, 0.36	**0.043**	0.01	0.001, 0.03	**0.036**
HOMA-IR	0.11	0.03, 0.19	**0.005**	0.04	**− **0.03, 0.12	0.306	0.03	**− **0.04, 0.11	0.383
MATSUDA index	**− **0.38	**− **0.56, **− **0.19	** < 0.001**	**− **0.19	**− **0.37, **− **0.02	**0.024**	**− **0.18	**− **0.35, **− **0.01	**0.038**
ISSI-2	**− **0.06	**− **0.11, **− **0.02	**0.009**	**− **0.03	**− **0.08, 0.02	0.226	**− **0.03	**− **0.08, 0.02	0.284
AUC_ins/glu_	0.01	0.006, 0.03	**0.002**	0.09	**− **0.007, 0.01	0.070	0.08	**− **0.01, 0.02	0.106
HOMA-B	1.59	0.41, 2.76	**0.008**	0.57	**− **0.57, 1.72	0.325	0.49	**− **0.67, 1.65	0.409
CRP at 1-year pp	*Insulin resistance and secretion variables at 1-year postpartum*								
Weight (kg)^bc^	1.59	0.99, 2.19	** < 0.001**	1.58	0.98, 2.19	** < 0.001**	NA	NA	NA
Body fat (kg)^c^	1.23	0.84, 1.61	** < 0.001**	0.25	0.13, 0.36	** < 0.001**	NA	NA	NA
MetS-BMI, yes^a^	1.20	1.08, 1.33	** < 0.001**	1.12	0.98, 1.27	0.073	1.09	0.95, 1.24	0.198
MetS-WC, yes^a^	1.15	1.05, 1.26	**0.002**	1.09	0.98, 1.21	0.098	1.07	0.96, 1.18	0.183
Fasting glucose (mmol/l)	0.02	0.003, 0.05	**0.027**	0.01	**− **0.009, 0.04	0.211	0.02	**− **0.01, 0.045	0.232
HbA1c (%)	0.002	**− **0.02, 0.03	0.808	**− **0.07	**− **0.03, 0.02	0.546	**− **0.01	**− **0.04, 0.01	0.364
HOMA-IR	0.19	0.09, 0.28	** < 0.001**	0.05	**− **0.03, 0.13	0.238	0.03	**− **0.06, 0.12	0.546
MATSUDA index	**− **0.24	**− **0.36, **− **0.12	** < 0.001**	**− **0.07	**− **0.18, 0.04	0.214	**− **0.04	**− **0.16, 0.07	0.439
ISSI-2	**− **0.05	**− **0.08, **− **0.01	**0.014**	**− **0.01	**− **0.05, 0.02	0.547	**− **0.07	**− **0.49, 0.35	0.739
AUC_ins/glu_	0.02	0.008, 0.03	**0.001**	0.07	0.03, 0.18	0.195	0.006	**− **0.006, 0.01	0.322
HOMA-B	2.13	0.85, 3.40	**0.001**	0.20	**− **0.95, 1.35	0.729	**− **0.20	**− **1.39, 0.97	0.726

CRP denotes C-reactive protein; GA denotes gestational age; MetS denote metabolic syndrome; BMI denotes Body Mass Index; Mets-WC denotes metabolic syndrome based on waist circumference; pp denotes postpartum; NA denoted not applicable; HbA1c denotes glycated hemoglobin; HOMA-IR denotes Homeostatic Model Assessment for Insulin Resistance; ISSI-2 denotes; Insulin Secretion-Sensitivity Index-2; AUCins/glu denotes Area under the Curve; HOMA-B denotes HOMA of b-cell index.

Model 1: Adjusted for group allocation. Model 2: Adjusted for group allocation, gestational age, age, previous history of GDM, family history of GDM, breastfeeding, weight at prediction time point. Model 3: adjusted for group allocation, gestational age, age, previous history of GDM, family history of GDM, breastfeeding, body fat at the prediction time point.

^a^Estimates are from logistic regression analyses (Odds ratio and 95% CI). MetS-WC is not relevant in pregnancy and too early to assess in the early pp

For these variables, the associations were not adjusted for ^b^weight or ^c^body fat (additionally adjusted for age).

Bold p-values indicate significant p-values

In an additional analysis, we investigated the longitudinal associations between CRP at 28–32 GA and at 6–8 weeks postpartum with metabolic health outcomes at 1 year postpartum independent of the medical history confounders and of fat-free mass instead of fat mass (at the prediction time point) and found similar results (Additional file 1: Table S3).

We also investigated the longitudinal associations between IL-6 and TNF-α at 28–32 weeks GA and at 6–8 weeks postpartum with metabolic health at 1 year postpartum (Additional file 1: Table S4). IL-6 was not related to metabolic health outcomes except for two associations: IL-6 at 28–32 weeks GA was associated with increased ISSI-2 whereas IL-6 at 6–8 weeks postpartum was associated with increased AUCins/glu at 1 year postpartum. TNF-α both 28–32 weeks GA and at 6–8 weeks postpartum was not associated with any of the metabolic health outcomes at 1 year postpartum.

## Discussion

We found prospective associations between CRP in the perinatal period and adverse metabolic outcomes at 1 year postpartum in women with GDM. CRP during pregnancy and in the early postpartum predicted higher weight, body fat, VAT, higher prevalence of the MetS and increased insulin resistance (HOMA-IR, MATSUDA) at 1 year postpartum. These associations, particularly CRP during pregnancy, were independent of medical history confounders related to diabetes risk including GA, age, previous history of GDM, family history of diabetes as well as breastfeeding and weight or changes in weight. For VAT, insulin resistance and MetS, these associations were even independent of medical history confounders and of body fat. CRP also predicted an increased absolute insulin secretion (HOMA-B, AUC_ins/glu_) at 1 year postpartum, which was independent of these confounders and of weight. However, it predicted a reduction in insulin resistance-adjusted insulin secretion (ISSI-2). These results were similar when we further adjusted for fat-free mass and results in the cross-sectional analyses were similar.

CRP during pregnancy and in the early postpartum period was related to increased weight, total and central body fat at 1 year postpartum. Our finding suggests an effect or a link between CRP with obesity in women with GDM. These results agree with existing cross-sectional studies in women with previous GDM [34, 35]. Importantly, the relationship with body fat remained after adjusting for weight and the one with VAT remained significant even after adjusting for total body fat. This is new for pregnancy and in line with data suggesting that VAT has an impact on inflammation that is beyond body fat.

CRP in pregnancy and in the early postpartum predicted higher insulin resistance: both higher hepatic insulin resistance (HOMA-IR) and lower whole body insulin sensitivity (MATSUDA) at 1-year postpartum. These results were independent of weight or body fat or changes in these parameters, indicating that CRP may independently play a role in the progression to insulin-resistant states. Our results were, not significant when we adjusted for VAT (data not shown), underlining that VAT, might play a role in the link between inflammation, insulin resistance and adverse metabolic health. Other studies found cross-sectional associations between CRP and hepatic insulin resistance (HOMA-IR) in GDM women [15, 16, 34], but they did not investigate the role of weight or body fat.

CRP is linked to hepatic insulin resistance through its involvement in impairing insulin signaling in the liver [11]. CRP is stimulated by IL-1β, TNF-α and IL-6 in the liver [8], which act together to activate serine and threonine kinases to suppress insulin signal transduction and thus promote insulin resistance. Despite the inter-relationship between IL-6, TNF-α and CRP, this study neither found similar associations between IL-6 and TNF-α with insulin resistance nor with other metabolic outcomes. This suggest that CRP is either a robust marker, particularly in the perinatal period, or it might play an independent role as it can have direct effects on glucose homeostasis [13]. IL-1β is probably a stronger stimulant of CRP compared to IL-6 and TNF-α. As cytokines act on the tissue level, their concentrations are often not reflected by the levels measured in the circulation.

In addition, CRP in the perinatal period predicted higher absolute insulin secretion (AUCins/glu, HOMA-B), but lower insulin resistance-adjusted insulin secretion (ISS1-2) at 1 year. The prediction of absolute insulin secretion by CRP is most likely related to the observed increase in insulin resistance, which was independent of weight and partly of body fat. However, it predicted a reduction in the relative or insulin-resistance-adjusted insulin secretion. We are not aware of other studies that investigated the relationship between CRP and insulin secretion in women with GDM, which can serve as a relatively homogenous model of metabolically high-risk subjects. The prospective association between CRP and increased absolute insulin secretion reflect a compensatory response of islet beta-cells related to the decreased insulin action. As it predicted a reduced insulin secretion independent of insulin resistance, CRP might be associated with or lead to reduced islet beta-cell function in women after GDM.

The increase in CRP during acute or chronic inflammation could affect islet cell viability to cause insulin secretory dysfunction through an effect on local circulation [13]. Although we did not investigate IL-1β, metabolic stress could induce IL-1β production from islet cells to contribute to impaired insulin secretion and might potentially explain why CRP predicted lower ISSI-2 in our study. Alternatively, the chronic increase in insulin resistance could lead to an overstimulation, but long-term exhaustion of the pancreatic beta-cells. The latter hypothesis might explain why our longitudinal and cross-sectional results were similar.

To test the robustness of our findings, we also confirmed the associations between CRP and metabolic outcomes during pregnancy and in the postpartum in cross-sectional analyses. CRP during pregnancy correlated with increased weight and body fat, as well as insulin resistance (HOMA-IR) and absolute secretion (HOMA-B), partly independent of weight. Similar results were observed in the postpartum, where in addition the relationship between CRP and reduced ISSI-2 was confirmed.

The strengths of this study include its prospective design and longitudinal follow-up up to 1-year postpartum of a homogenous sample of women at high metabolic risk. Insulin resistance and secretion were measured by several validated indices. In addition to CRP, the proinflammatory cytokines TNF-α and IL-6 were also tested to better understand the process linking CRP to adverse metabolic health outcomes. We also investigated if associations between CRP and metabolic outcomes were independent of weight or body fat. Despite this, the lack of data on IL-1β and IL-1RA potentially limit the understanding of our results. The lack of a comparable control group, i.e., women without GDM is a major limitation. The prediction of future metabolic risk by CRP might in part be accounted by its role as a proxy of insulin resistance in pregnancy, but it still offers the advantage of being a useful marker in pregnancy that does not need to be measured in a fasting state. Although the accuracy or usefulness of HbA1c in pregnancy has been controversial [36], it can be useful to identify women at risk for future GDM [37], perinatal complications [38], and future type 2 diabetes [39]. The slightly reduced number of women at 1‐year follow‐up is a further limitation, but this represents less than 16% of the entire population. Importantly, a longer follow-up period including cardiovascular events may be helpful. Although breastfeeding is known to be associated with increased insulin sensitivity, improved insulin secretion, and postpartum weight loss, the observed associations in our study were independent of the effect of breastfeeding. In addition, we pooled both intervention and control participants together to increase the sample size, since the value of predictors and outcomes and the effect sizes were similar in both groups. We adjusted for group allocation in all analyses.

## Conclusions

In this cohort of women with GDM followed during pregnancy up 1 year postpartum, CRP in the perinatal period predicted a more adverse metabolic profile including insulin resistance, decreased adjusted insulin secretion and metabolic syndrome that was largely independent of GA, age, previous history of GDM, family history of diabetes and weight and in part of body fat. Therefore, CRP may be a novel biomarker in patients with GDM. If our results can be confirmed, CRP could be used to risk stratify women with GDM earlier for targeted interventions. It remains to be shown whether anti-inflammatory treatment that reduces CRP can improve outcomes in these patients.

### Supplementary Information

**Additional file 1: Table S1.** Differences in inflammatory markers during pregnancy and postpartum according to pre-pregnancy BMI groups. **Table S2.** Metabolic health outcomes during pregnancy and postpartum according to pre-pregnancy BMI groups. **Table S3.** Longitudinal associations between CRP during pregnancy and at 6–8 weeks postpartum with metabolic health outcomes at 1-year postpartum after adjustment for confounders and fat-free mass. **Table S4.** Longitudinal associations between IL-6 and TNF-alpha during pregnancy and at 6–8 weeks postpartum with metabolic control variables at 1-year postpartum. **Table S5.** Longitudinal associations between CRP during pregnancy and at 6–8 weeks postpartum with metabolic health outcomes at 1 year postpartum.

## Data Availability

The datasets generated and/or analysed during the current study are not publicly available as they are clinical data but are available from the corresponding author on reasonable request.

## Abbreviations

GDM: Gestational diabetes mellitus
CVD: Cardiovascular disease
CRP: C-reactive protein
TNF-α: Tumor necrosis factor-alpha
MetS: Metabolic syndrome
GA: Gestational age
GWG: Gestational weight gain
IOM: Institute of Medicine
VAT: Visceral adipose tissue
DXA: Dual-Energy X-ray Absorptiometry
oGTT: Oral glucose tolerance test
HOMA-IR: The Homeostatic Model Assessment for Insulin Resistance
ISSI-2: Insulin Secretion-Sensitivity Index-2
AUC: Area under the Curve
HOMA-B: HOMA of b-cell index

## References

[1] Kramer CK, Campbell S, Retnakaran R (2019). Gestational diabetes and the risk of cardiovascular disease in women: a systematic review and meta-analysis. Diabetologia.

[2] Gunderson EP, Sun B, Catov JM, Carnethon M, Lewis CE, Allen NB (2021). Gestational diabetes history and glucose tolerance after pregnancy associated with coronary artery calcium in women during midlife the CARDIA Study. Circulation.

[3] Buchanan TA, Xiang AH (2005). Gestational diabetes mellitus. J Clin Invest.

[4] Pickut JC (2004). Inflammation and activated innate immunity in the pathogenesis of type 2 diabetes. Diabetes Care.

[5] Ridker PM. High-Sensitivity C-Reactive Protein. 2016. http://www.acc.org/jacc-journals-cme

[6] Devaraj S, Singh U, Jialal I (2009). Human C-reactive protein and the metabolic syndrome. Curr Opin Lipidol.

[7] Larsen CM, Faulenbach M, Vaag A, Vølund A, Ehses JA, Seifert B, et al. Interleukin-1-Receptor Antagonist in Type 2 Diabetes Mellitus. N Engl J Med. 2007: 356. www.nejm.org 10.1056/NEJMoa065213 17429083

[8] Gabay C, Kushner I (1999). Acute-phase proteins and other systemic responses to inflammation. N Engl J Med.

[9] Missel AL, Saslow LR, Griauzde DH, Marvicsin D, Sen A, Richardson CR (2021). Association between fasting insulin and C-reactive protein among adults without diabetes using a two-part model: NHANES 2005–2010. Diabetol Metab Syndr.

[10] Pradhan AD, Cook NR, Buring JE, Manson JAE, Ridker PM (2003). C-reactive protein is independently associated with fasting insulin in nondiabetic women. Arterioscler Thromb Vasc Biol.

[11] Xi L, Xiao C, Bandsma RHJ, Naples M, Adeli K, Lewis GF (2011). C-reactive protein impairs hepatic insulin sensitivity and insulin signaling in rats: Role of mitogen-activated protein kinases. Hepatology.

[12] Rohm TV, Meier DT, Olefsky JM, Donath MY (2022). Inflammation in obesity, diabetes, and related disorders. Immunity.

[13] Chan PC, Wang YC, Chen YL, Hsu WN, Tian YF, Hsieh PS (2017). Importance of NADPH oxidase-mediated redox signaling in the detrimental effect of CRP on pancreatic insulin secretion. Free Radic Biol Med.

[14] Fizelova M, Jauhiainen R, Kangas AJ, Soininen P, Ala-Korpela M, Kuusisto J (2017). Differential associations of inflammatory markers with insulin sensitivity and secretion: the prospective METSIM study. J Clin Endocrinol Metab.

[15] McLachlan KA, O’Neal D, Jenkins A, Alford FP (2006). Do adiponectin, TNFα, leptin and CRP relate to insulin resistance in pregnancy? Studies in women with or without gestational diabetes, during and after pregnancy. Diabetes Metab Res Rev.

[16] Carpenter MW (2007). Gestational diabetes, pregnancy hypertension, and late vascular disease. Diabetes Care.

[17] Edalat B, Sharifi F, Badamchizadeh Z, Hossein-Nezhad A, Larijani B, Mirarefin M (2013). Association of metabolic syndrome with inflammatory mediators in women with previous gestational diabetes mellitus. J Diabetes Metab Disord.

[18] Can B, Tutuncu Y, Can B, Keskin H, Bekpinar S, Dinccag N (2022). Inflammatory markers are associated with the progression of gestational diabetes to metabolic syndrome. J Obstet Gynaecol.

[19] Schulze F, Wehner J, Kratschmar DV, Makshana V, Meier DT, Häuselmann SP (2020). Inhibition of IL-1beta improves Glycaemia in a Mouse Model for Gestational Diabetes. Sci Rep.

[20] Horsch A, Gilbert L, Lanzi S, Gross J, Kayser B, Vial Y (2018). Improving cardiometabolic and mental health in women with gestational diabetes mellitus and their offspring: Study protocol for MySweetHeart Trial, a randomised controlled trial. BMJ Open.

[21] Metzger BE (2010). International Association of Diabetes and Pregnancy Study Groups recommendations on the diagnosis and classification of hyperglycemia in pregnancy. Diabetes Care.

[22] Elsayed NA, Aleppo G, Aroda VR, Bannuru RR, Brown FM, Bruemmer D (2023). 15. Management of diabetes in pregnancy: standards of care in diabetes—2023. Diabetes Care.

[23] Blumer I, Hadar E, Hadden DR, Jovanovič L, Mestman JH, Murad MH (2013). Diabetes and pregnancy: an endocrine society clinical practice guideline. J Clin Endocrinol Metab.

[24] Institute of Medicine (IOM). Weight gain during pregnancy: reexamining the guidelines. Committee to Reexamine IOM Pregnancy Weight Guidelines. Washington, DC; 2009.

[25] Arditi C, Puder J, Vial Y, Hagon-Traub I, Burnand B (2018). Grossesse et diabète Prise en charge multidisciplinaire du diabète: recommandations pour la pratique clinique. Revue Medicale Suisse..

[26] Gilbert L, Quansah DY, Arhab A, Schenk S, Gross J, Lanzi S (2023). Effect of the MySweetheart randomized controlled trial on birth, anthropometric and psychobehavioral outcomes in offspring of women with GDM. Front Endocrinol.

[27] Kyle UG, Genton L, Slosman DO, Pichard C (2001). Fat-free and fat mass percentiles in 5225 healthy subjects aged 15 to 98 years. Nutrition.

[28] Alberti KGMM, Eckel RH, Grundy SM, Zimmet PZ, Cleeman JI, Donato KA (2009). Harmonizing the metabolic syndrome: a joint interim statement of the international diabetes federation task force on epidemiology and prevention; National heart, lung, and blood institute; American heart association; World heart federation International. Circulation.

[29] Jeppsson JO, Kobold U, Barr J, Finke A, Hoelzel W, Hoshino T (2002). Approved IFCC reference method for the measurement of HbA1c in human blood. Clin Chem Lab Med.

[30] Matthews DR, Hosker JP, Rudenski AS, Naylor BA, Treacher DF, Turner RC (1985). Homeostasis model assessment: insulin resistance and β-cell function from fasting plasma glucose and insulin concentrations in man. Diabetologia.

[31] Matsuda M, DeFronzo RA (1999). Insulin sensitivity indices obtained from oral glucose tolerance testing: comparison with the euglycemic insulin clamp. Diabetes Care.

[32] Retnakaran R, Qi Y, Sermer M, Connelly PW, Hanley AJG, Zinman B (2010). β-cell function declines within the first year postpartum in women with recent glucose intolerance in pregnancy. Diabetes Care.

[33] Song Y, Manson JE, Tinker L, Howard BV, Kuller LH, Nathan L (2007). Insulin sensitivity and insulin secretion determined by homeostasis model assessment and risk of diabetes in a multiethnic cohort of women: the women’s health initiative observational study. Diabetes Care.

[34] Di Benedetto A, Russo GT, Corrado F, Di Cesare E, Alessi E, Nicocia G (2005). Inflammatory markers in women with a recent history of gestational diabetes mellitus. J Endocrinol Invest.

[35] Retnakaran R, Hanley AJG, Raif N, Connelly PW, Sermer M, Zinman B (2003). C-reactive protein and gestational diabetes: The central role of maternal obesity. J Clin Endocrinol Metab.

[36] Elsayed NA, Aleppo G, Aroda VR, Bannuru RR, Brown FM, Bruemmer D (2023). 15. Management of diabetes in pregnancy: standards of care in diabetes—2023. Diabetes Care.

[37] Kattini R, Hummelen R, Kelly L (2020). Early gestational diabetes mellitus screening with glycated hemoglobin: a systematic review. J Obst Gynaecol Canada..

[38] Antoniou MC, Gilbert L, Gross J, Rossel JB, Fischer Fumeaux CJ, Vial Y (2019). Potentially modifiable predictors of adverse neonatal and maternal outcomes in pregnancies with gestational diabetes mellitus: Can they help for future risk stratification and risk-adapted patient care?. BMC Pregnancy Childbirth.

[39] Kawasaki M, Arata N, Sakamoto N, Osamura A, Sato S, Ogawa Y (2020). Risk factors during the early postpartum period for type 2 diabetes mellitus in women with gestational diabetes. Endocr J.

