# Supplementary material for: C-reactive protein during pregnancy and in the early postpartum predicts adverse metabolic health outcomes at 1 year postpartum in women with gestational diabetes
Source: Cardiovasc Diabetol. 2023 Oct 27;22:291. doi: 10.1186/s12933-023-02034-9 (PMC10612338; doi:10.1186/s12933-023-02034-9)
Supplement: Supplementary file 1 — Additional file 1: Table S1. Differences in inflammatory markers during pregnancy and postpartum according to pre-pregnancy BMI groups. Table S2. Metabolic health outcomes during pregnancy and postpartum according to pre-pregnancy BMI groups. Table S3. Longitudinal associations between CRP during pregnancy and at 6–8 weeks postpartum with metabolic health outcomes at 1-year postpartum after adjustment for confounders and fat-free mass. Table S4. Longitudinal associations between IL-6 and TNF-alpha during pregnancy and at 6–8 weeks postpartum with metabolic control variables at 1-year postpartum. Table S5. Longitudinal associations between CRP during pregnancy and at 6–8 weeks postpartum with metabolic health outcomes at 1 year postpartum. [file 12933_2023_2034_MOESM1_ESM.docx]

**Supplementary tables**

Table 1: Differences in inflammatory markers during pregnancy and postpartum according to pre-pregnancy BMI groups

|  | **Pre-pregnancy BMI categories** | | | | **P-value** |
| --- | --- | --- | --- | --- | --- |
| Variable | **All**  **(n=211)** | **Normal**  **(n=108)** | **Overweight**  **(n=61)** | **Obese**  **(n=42)** |  |
|  |  | **Mean±SD** | **Mean±SD** | **Mean±SD** |  |
| **28-32 weeks GA (n=211)** |  |  |  |  |  |
| CRP (mg/l) | 4.5±3.7 | 3.2±2.8 | 4.9±3.7 | 7.0±4.1 | **<0.001** |
| Median (IQR) | 3.2 (4.5) | 2.2 (2.3) | 3.7 (4.4) | 6.1 (5.3) |  |
| IL-6 (pg/ml) | 0.99±1.3 | 1.10±1.8 | 0.84±0.7 | 0.94±0.5 | 0.500 |
| Median (IQR) | 0.73 (0.67) | 0.76 (0.72) | 0.64 (0.46) | 0.82 (0.64) |  |
| TNF-α (pg/ml) | 0.72±0.7 | 0.79±0.88 | 0.70±0.7 | 0.59±0.34 | 0.334 |
| Median (IQR) | 0.62 (0.54) | 0.63 (0.61) | 0.61 (0.57) | 0.62 (0.37) |  |
| **At 6-8 weeks pp (n=191)** |  |  |  |  |  |
| CRP (mg/l) | 3.4±3.2 | 2.6±2.9 | 3.2±2.7 | 5.3±3.8 | **<0.001** |
| Median (IQR) | 2.2 (2.8) | 1.6 (2.0) | 2.3 (2.4) | 3.7 (7.1) |  |
| IL-6 (pg/ml) | 0.72±1.0 | 0.76±1.3 | 0.63±0.4 | 0.75±0.59 | 0.738 |
| Median (IQR) | 0.5 (0.51) | 0.47 (0.45) | 0.55 (0.45) | 0.60 (0.64) |  |
| TNF-α (pg/ml) | 0.61±0.8 | 0.68±1.1 | 0.54±0.2 | 0.55±0.3 | 0.482 |
| Median (IQR) | 0.51 (0.35) | 0.48 (0.32) | 0.53 (0.31) | 0.54 (0.45) |  |
| **At 1-year pp (n=157)** |  |  |  |  |  |
| CRP (mg/l) | 3.0±3.8 | 1.9±2.4 | 2.9±3.2 | 5.9±5.7 | **<0.001** |
| Median (IQR) | 1.5 (2.9) | 1.2 (1.9) | 1.8 (2.3) | 4.4 (7.5) |  |
| IL-6 (pg/ml) | 0.91±1.4 | 0.83±1.1 | 1.10±2.2 | 0.84±0.6 | 0.596 |
| Median (IQR) | 0.57 (0.56) | 0.57 (0.50) | 0.46 (0.56) | 0.71 (0.80) |  |
| TNF-α (pg/ml) | 0.79±1.1 | 0.74±0.9 | 0.83±1.4 | 0.87±0.83 | 0.822 |
| Median (IQR) | 0.52 (0.35) | 0.49 (0.38) | 0.50 (0.33) | 0.64 (0.53) |  |

GA denotes gestational age; BMI denotes body mass index; SD denotes standard deviaton; pp denotes postpartum; CRP denotes C-reactive protein; IL-6 denotes interleukin 6; TNF-α denotes Tumor necrosis factor alpha; IQR denotes Interquartile range

Data is presented as mean±standard deviation.

P-values are derived from ANOVA

Table 2: Metabolic health outcomes during pregnancy and postpartum according to pre-pregnancy BMI groups

|  |  | **Pre-pregnancy BMI categories** | | | **P-value** |
| --- | --- | --- | --- | --- | --- |
| Variable | **All**  **(n=211)** | **Normal**  **(n=108)** | **Overweight**  **(n=61)** | **Obese**  **(n=42)** |  |
|  |  | **Mean±SD** | **Mean±SD** | **Mean±SD** |  |
| **28-32 weeks GA** |  |  |  |  |  |
| Weight at the first GDM visit (kg) | 79.9±14.6 | 70.3±8.7 | 83.6±7.7 | 95.6±15.3 | **<0.001** |
| Total gestational weight gain (kg) | 11.9±9.2 | 15.3±6.2 | 10.9±6.3 | 6.2±13.5 | **<0.001** |
| Rate of weight gain per week (kg) | 0.3±0.2 | 0.38±0.1 | 0.28±0.2 | 0.16±0.3 | **<0.001** |
| Body fat (kg) | 32.1±9.4 | 25.6±5.4 | 34.4±4.9 | 42.7±8.8 | **<0.001** |
| MetS-BMI, yes (n, %) | 40 (18.9) | 6 (5.9) | 15 (25.0) | 19 (38.8) | **<0.001** |
| Fasting glucose at the first GDM visit (mmol/l) | 4.9±0.5 | 4.8±0.5 | 5.1±0.5 | 5.3±0.6 | **0.001** |
| 2h glucose at the first GDM visit (mmol/l) | 8.1±20. | 8.2±1.6 | 8.0±1.4 | 7.8±1.9 | 0.555 |
| HbA1c (%) at the first GDM visit | 5.3±0.3 | 5.2±0.3 | 5.3±0.2 | 5.4±0.3 | **0.002** |
| HOMA-IR at first GDM visit | 3.8±2.5 | 3.0±2.8 | 4.1±2.2 | 5.1±1.7 | **<0.001** |
| HOMA-B | 61.72±32.0 | 49.4±27.2 | 65.5±33.5 | 82.2±33.5 | **<0.001** |
| **At the 6-8 weeks pp** |  |  |  |  |  |
| Weight (kg) | 74.0±14.9 | 64.2±8.5 | 77.2±7.9 | 91.0±14.6 | **<0.001** |
| Body fat (kg) | 28.4±9.7 | 21.7±5.2 | 30.4±5.5 | 40.1±9.1 | **<0.001** |
| BMI (kg/m^2^) | 27.5±5.2 | 23.9±2.8 | 28.4±2.3 | 34.1±4.4 | **<0.001** |
| MetS-BMI, yes (n, %) | 32 (15.2) | 6 (5.9) | 9 (15.0) | 17 (34.7) | **<0.001** |
| MetS-WC, yes (n, %) | 47 (22.3) | 14 (13.7) | 14 (23.3) | 19 (38.8) | **0.003** |
| Fasting glucose (mmol/l | 5.1±0.7 | 4.9±0.6 | 5.1±0.6 | 5.4±0.9 | **0.004** |
| 2h glucose (mmol/l) | 5.4±1.4 | 5.2±1.5 | 5.5±1.3 | 5.4±1.1 | 0.453 |
| Glucose tolerance status, (yes) (n, %) |  |  |  |  |  |
| Normal | 163 (85.3) | 75 (88.2) | 47 (81.0) | 41 (85.4) | 0.213 |
| Prediabetes | 27 (14.2) | 10 (11.8) | 11 (18.9) | 6 (12.5) |  |
| Diabetes | 1 (0.5) | 0 | 0 | 1 (2.1) |  |
| HbA1c (%) | 5.3±0.3 | 5.3±0.4 | 5.2±0.3 | 5.2±0.4 | 0.645 |
| HOMA-IR | 2.2±1.9 | 1.6±1.2 | 2.3±2.1 | 3.4±2.5 | **<0.001** |
| MATSUDA index | 7.0±3.7 | 8.2±3.9 | 6.5±3.4 | 4.2±1.4 | **<0.001** |
| ISSI-2 | 2.53±0.97 | 2.84±1.11 | 2.33±0.71 | 2.05±0.56 | **0.003** |
| AUC_ins/glu_ | 0.43±0.21 | 0.37±0.15 | 0.44±0.24 | 0.56±0.24 | **<0.001** |
| HOMA-B | 31.7±26.6 | 22.7±16.4 | 34.2±30.1 | 46.7±32.0 | **<0.001** |
| **At the 1-year pp** |  |  |  |  |  |
| Weight (kg) | 72.4±16.2 | 61.1±8.5 | 77.0±8.6 | 92.2±15.0 | **<0.001** |
| Body fat (kg) | 26.6±10.6 | 19.2±5.5 | 29.2±5.2 | 39.7±10.2 | **<0.001** |
| Visceral adipose tissue (kg) | 0.59±0.4 | 0.28±0.3 | 0.69±0.3 | 1.1±0.3 | **<0.001** |
| BMI (kg/m^2^) | 26.8±4.5 | 22.7±2.9 | 28.3±2.4 | 34.5±4.5 | **<0.001** |
| MetS-BMI, yes | 53 (25.1) | 13 (12.7) | 13 (21.6) | 27 (55.1) | **<0.001** |
| MetS-WC, yes | 70 (33.2) | 19 (18.6) | 22 (36.7) | 29 (59.2) | **<0.001** |
| Fasting glucose (mmol/l | 5.4±0.6 | 5.2±0.5 | 5.5±0.8 | 5.5±0.5 | **0.002** |
| 2h glucose (mmol/l) | 5.9±1.6 | 5.5±1.6 | 6.2±1.8 | 6.3±1.4 | **0.020** |
| Glucose tolerance status, (yes) (n, %) |  |  |  |  |  |
| Normal | 97 (61.8) | 47 (71.2) | 32 (61.5) | 18 (46.1) | **0.002** |
| Prediabetes | 55 (35.0) | 18 (27.3) | 17 (32.7) | 20 (51.3) |  |
| Diabetes | 5 (3.2) | 1 (1.5) | 3 (5.8) | 1 (2.6) |  |
| HbA1c (%) | 5.3±0.5 | 5.2±0.4 | 5.3±0.7 | 5.5±0.7 | **0.010** |
| HOMA-IR | 3.3±2.3 | 2.0±1.5 | 3.5±2.2 | 5.3±2.6 | **<0.001** |
| MATSUDA index | 4.7±2.8 | 6.3±2.9 | 3.9±2.0 | 2.4±0.7 | **<0.001** |
| ISSI-2 | 2.09±0.81 | 2.46±0.88 | 1.84±0.61 | 1.60±0.48 | **<0.001** |
| AUC_ins/glu_ | 0.54±0.28 | 0.43±0.16 | 0.54±0.26 | 0.79±0.36 | **<0.001** |
| HOMA-B | 45.2±38.1 | 29.9±16.6 | 47.3±27.0 | 73.5±38.1 | **<0.001** |

CRP denotes C-reactive protein; GA denotes gestational age; MetS denote metabolic syndrome; BMI denotes Body Mass Index; Mets-WC denotes metabolic syndrome based on waist circumference; MetS-BMI denotes metabolic syndrome based on BMI; pp denotes postpartum; NA denoted not applicable; HbA1c denotes glycated hemoglobin; HOMA-IR denotes Homeostatic Model Assessment for Insulin Resistance; ISSI-2 denotes; Insulin Secretion-Sensitivity Index-2; AUC denotes Area under the Curve; HOMA-B denotes HOMA of b-cell index

Data is presented as mean±standard deviation unless otherwise stated.

P-values are derived from ANOVA (continuous variables) or Chi-square test (categorical variables)

Table 3: Longitudinal associations between CRP during pregnancy and at 6-8 weeks postpartum with metabolic health outcomes at 1-year postpartum after adjustment for confounders and fat-free mass

| Variable | **β-coefficient** | **95% CI** | **P value** |
| --- | --- | --- | --- |
| **CRP at 28-32 weeks GA** |  |  |  |
| Visceral adipose tissue (kg) | 0.03 | 0.01, 0.05 | 0.001 |
| MetS-WC, yes^a^ | 1.20 | 1.09, 1.33 | **<0.001** |
| Fasting glucose (mmol/l) | 0.02 | -0.01, 0.04 | **0.216** |
| 2h glucose (mmol/l) | 0.005 | -0.07, 0.09 | 0.891 |
| HbA1c (%) | 0.004 | -0.02, 0.03 | 0.730 |
| HOMA-IR | 0.11 | 0.01, 0.20 | **0.023** |
| MATSUDA index | -0.16 | -0.29, -0.03 | **0.015** |
| ISSI-2 | -0.02 | -0.06, 0.009 | 0.134 |
| AUC_ins/glu_ | 0.01 | -0.002, 0.02 | 0.103 |
| HOMA-B | 1.41 | 0.10, 2.71 | **0.034** |
| **CRP at 6-8 weeks pp** |  |  |  |
| Visceral adipose tissue (kg) | 0.04 | 0.02, 0.06 | **<0.001** |
| MetS-WC, yes^a^ | 1.14 | 1.03, 1.26 | **0.011** |
| Fasting glucose (mmol/l) | -0.003 | -0.03, 0.03 | 0.985 |
| 2h glucose (mmol/l) | -0.14 | -0.10, 0.07 | 0.746 |
| HbA1c (%) | -0.01 | -0.03, 0.01 | 0.306 |
| HOMA-IR | 0.15 | 0.05, 0.24 | **0.002** |
| MATSUDA index | -0.18 | -0.33, -0.02 | **0.021** |
| ISSI-2 | -0.04 | -0.08, 0.01 | 0.119 |
| AUC_ins/glu_ | 0.01 | 0.007, 0.02 | **0.039** |
| HOMA-B | 2.07 | 0.88, 3.25 | **0.001** |

CRP denotes C-reactive protein; GA denotes gestational age; MetS denote metabolic syndrome; BMI denotes Body Mass Index; Mets-WC denotes metabolic syndrome based on waist circumference; pp denotes postpartum; NA denoted not applicable; HbA1c denotes glycated hemoglobin; HOMA-IR denotes Homeostatic Model Assessment for Insulin Resistance; ISSI-2 denotes; Insulin Secretion-Sensitivity Index-2; AUC denotes Area under the Curve; HOMA-B denotes HOMA of b-cell index. Results were adjusted for group allocation, gestational age, age, previous history of GDM, family history of GDM, and fat free mass at the prediction time point. ^a^Estimates are from logistic regression analyses (Odds ratio and 95% CI)

Table 4: Longitudinal associations between IL-6 and TNF-alpha during pregnancy and at 6-8 weeks postpartum with metabolic control variables at 1-year postpartum

| Variable | **β-coefficient** | **95% CI** | **P value** |
| --- | --- | --- | --- |
| **IL-6** |  |  |  |
| *28-32 weeks GA* |  |  |  |
| HOMA-IR | -0.08 | -0.33-0.18 | 0.549 |
| MATSUDA index | 0.30 | -0.49-1.10 | 0.451 |
| ISSI-2 | 0.26 | 0.04-0.48 | **0.022** |
| AUC_ins/glu_ | 0.04 | -0.04-0.12 | 0.338 |
| HOMA-B | -0.85 | -4.30-2.59 | 0.624 |
| *At 6-8 weeks postpartum* |  |  |  |
| HOMA-IR | 0.08 | -0.25-0.42 | 0.620 |
| MATSUDA index | -0.29 | -0.74-0.14 | 0.188 |
| ISSI-2 | 0.03 | -0.10-0.15 | 0.658 |
| AUC_ins/glu_ | 0.07 | 0.02-0.11 | **0.003** |
| HOMA-B | 0.79 | -3.44-5.04 | 0.710 |
| **TNF-alpha** |  |  |  |
| *28-32 weeks GA* |  |  |  |
| HOMA-IR | -0.24 | -0.76-0.28 | 0.368 |
| MATSUDA index | 0.57 | -0.29-1.45 | 0.191 |
| ISSI-2 | 0.23 | -0.01-0.47 | 0.068 |
| AUC_ins/glu_ | -0.01 | -0.10-0.07 | 0.724 |
| HOMA-B | -2.58 | -9.72-4.54 | 0.475 |
| *At 6-8 weeks postpartum* |  |  |  |
| HOMA-IR | -0.02 | -0.41-0.41 | 0.991 |
| MATSUDA index | -0.23 | -0.78-0.30 | 0.386 |
| ISSI-2 | 0.03 | -0.12-0.18 | 0.673 |
| AUC_ins/glu_ | 0.05 | -0.02-0.10 | 0.062 |
| HOMA-B | -2.10 | -5.42-5.00 | 0.936 |

IL-6 denotes interleukin 6; TNF-α denotes Tumor necrosis factor alpha; GA denotes gestational age; HOMA-IR denotes Homeostatic Model Assessment for Insulin Resistance; ISSI-2 denotes; Insulin Secretion-Sensitivity Index-2; AUC denotes Area under the Curve; HOMA-B denotes HOMA of b-cell index.

All data adjusted for group allocation and gestational age. P-values are derived from linear regression analysis.

Table 5: Longitudinal associations between CRP during pregnancy and at 6-8 weeks postpartum with metabolic health outcomes at 1-year postpartum

|  | **Model 1** | | | **Model 2** | | | **Model 3** | | |
| --- | --- | --- | --- | --- | --- | --- | --- | --- | --- |
| Variable | **β-coefficient** | **95% CI** | **P value** | **β-coefficient** | **95% CI** | **P value** | **β-coefficient** | **95% CI** | **P value** |
| **CRP at 28-32 weeks GA** |  |  |  |  |  |  |  |  |  |
| Weight (kg)^*#^ | 1.70 | 1.02, 2.38 | **<0.001** | 1.49 | 0.88, 2.10 | **<0.001** | NA | NA | NA |
| Body fat (kg)^#^ | 1.32 | 0.89, 1.75 | **<0.001** | 1.19 | 0.79, 1.58 | **<0.001** | NA | NA | NA |
| Visceral adipose tissue (kg) | 0.05 | 0.02, 0.07 | **<0.001** | 0.04 | 0.02, 0.06 | **0.001** | 0.04 | 0.02, 0.06 | **0.001** |
| MetS-WC, yes^a^ | 1.22 | 1.12, 1.34 | **<0.001** | 1.21 | 1.08, 1.35 | **0.001** | 1.22 | 1.09, 1.37 | **0.001** |
| Fasting glucose (mmol/l) | 0.03 | 0.001, 0.05 | **0.036** | 0.02 | -0.03, 0.05 | 0.083 | 0.03 | -0.06, 0.05 | 0.055 |
| 2h glucose (mmol/l) | 0.03 | -0.04, 0.11 | 0.435 | -0.007 | -0.08, 0.07 | 0.986 | 0.09 | -0.08, 0.07 | 0.981 |
| HbA1c (%) | 0.01 | -0.01, 0.04 | 0.326 | 0.006 | -0.02, 0.03 | 0.614 | 0.04 | -0.02, 0.03 | 0.743 |
| HOMA-IR | 0.20 | 0.10, 0.31 | **<0.001** | 0.16 | 0.07, 0.26 | **0.001** | 0.16 | 0.06, 0.26 | **0.001** |
| MATSUDA index | -0.28 | -0.42, -0.15 | **<0.001** | -0.20 | -0.33, -0.08 | **0.002** | -0.21 | -0.35, -0.08 | **0.002** |
| ISSI-2 | -0.05 | -0.09, -0.02 | **0.002** | -0.04 | -0.07, -0.005 | **0.025** | -0.04 | -0.08, -0.05 | **0.026** |
| AUC_ins/glu_ | 0.02 | 0.009, 0.03 | **0.002** | 0.01 | 0.004, 0.03 | **0.013** | 0.02 | 0.004, 0.03 | **0.014** |
| HOMA-B | 2.63 | 1.23, 4.03 | **0.016** | 2.15 | 0.86, 3.45 | **0.001** | 2.09 | 0.77, 3.42 | **0.002** |
| **CRP at 6-8 weeks pp** |  |  |  |  |  |  |  |  |  |
| Weight (kg) ^*#^ | 1.60 | 0.89, 2.31 | **<0.001** | 1.51 | 0.87, 2.15 | **<0.001** | NA | NA | NA |
| Body fat (kg)^#^ | 1.21 | 0.75, 1.67 | **<0.001** | 1.16 | 0.74, 1.58 | **0.001** | NA | NA | NA |
| Visceral adipose tissue (kg) | 0.06 | 0.04, 0.09 | **<0.001** | 0.06 | 0.40, 0.08 | **<0.001** | 0.06 | 0.03, 0.08 | **0.001** |
| MetS-WC, yes^a^ | 1.15 | 1.05, 1.26 | **0.003** | 1.17 | 1.05, 1.31 | **0.004** | 1.17 | 1.05, 1.31 | **0.004** |
| Fasting glucose (mmol/l) | 0.01 | -0.01, 0.04 | 0.379 | 0.009 | -0.02, 0.04 | 0.569 | 0.007 | -0.02, 0.03 | 0.634 |
| 2h glucose (mmol/l) | 0.02 | -0.06, 0.10 | 0.634 | 0.01 | -0.07, 0.09 | 0.810 | 0.007 | -0.07, 0.09 | 0.859 |
| HbA1c (%) | 0.04 | -0.02, 0.02 | 0.970 | -0.006 | -0.03, 0.01 | 0.609 | 0.009 | -0.03, 0.01 | 0.485 |
| HOMA-IR | 0.21 | 0.11, 0.31 | **<0.001** | 0.19 | 0.09, 0.29 | **0.001** | 0.19 | 0.08, 0.29 | **0.001** |
| MATSUDA index | -0.27 | -0.41, -0.11 | **0.001** | -0.27 | -0.43, -0.11 | **0.001** | -0.24 | -0.40, -0.08 | **0.003** |
| ISSI-2 | -0.06 | -0.10, -0.01 | **0.010** | -0.06 | -0.11, -0.01 | **0.011** | -0.05 | -0.10, -0.009 | **0.020** |
| AUC_ins/glu_ | 0.02 | 0.005, 0.03 | **0.007** | 0.02 | 0.004, 0.03 | **0.010** | 0.01 | 0.003, 0.003 | **0.017** |
| HOMA-B | 2.82 | 1.52, 4.12 | **0.001** | 2.60 | 1.35, 3.84 | **0.001** | 2.55 | 1.26, 3.83 | **0.001** |

CRP denotes C-reactive protein; GA denotes gestational age; MetS denote metabolic syndrome; BMI denotes Body Mass Index; Mets-WC denotes metabolic syndrome based on waist circumference; pp denotes postpartum; NA denoted not applicable; HbA1c denotes glycated hemoglobin; HOMA-IR denotes Homeostatic Model Assessment for Insulin Resistance; ISSI-2 denotes; Insulin Secretion-Sensitivity Index-2; AUC denotes Area under the Curve; HOMA-B denotes HOMA of b-cell index. Model 1: adjusted for group allocation. Model 2: Adjusted for group allocation, gestational age, age, previous history of GDM, family history of GDM, breastfeeding, change in weight between pregnancy and 1-year postpartum. Model 3: adjusted for group allocation, gestational age, age, previous history of GDM, family history of GDM, breastfeeding and change in body fat between pregnancy and 1-year postpartum. For these variables, the associations were not adjusted for *weight or ^#^body fat (only adjusted for age). ^a^Estimates are from logistic regression analyses (Odds ratio and 95% CI)
